# Effects of the Substrate on Interfacial Polymerization: Tuning the Hydrophobicity via Polyelectrolyte Deposition

**DOI:** 10.3390/membranes10100259

**Published:** 2020-09-26

**Authors:** Xin Liu, Ge Liu, Weiyi Li, Qinyu Wang, Baolin Deng

**Affiliations:** 1 School of Environmental Science and Engineering, Southern University of Science and Technology, Shenzhen 518055, China; liux5@sustech.edu.cn (X.L.); 11749132@mail.sustech.edu.cn (G.L.); qwang437@gatech.edu (Q.W.); 2 Department of Civil and Environmental Engineering, University of Missouri, Columbia, MO 65211, USA; DengB@missouri.edu

**Keywords:** interfacial polymerization, thin film composite membranes, substrate hydrophilicity, polyelectrolyte deposition, forward osmosis efficiency

## Abstract

Interfacial polymerization (IP) has been the key method for the fabrication of the thin film composite (TFC) membranes that are extensively employed in reverse osmosis (RO) and forward osmosis (FO). However, the role of the substrate surface hydrophilicity in the formation of the IP-film remains a controversial issue to be further addressed. This study characterized the IP films formed on a series of polyacrylonitrile (PAN) substrates whose hydrophilicities (from ~38 to ~93 degrees) were varied via different approaches, including the alkaline treatment and the deposition of various polycations. It was revealed that delamination could occur when the IP film was formed on a relatively hydrophilic surface; the integrity of the TFC membranes was substantially improved, owing to the modification of the polyelectrolyte deposition. On the other hand, the characterization indicated that the TFC membrane could have an enhanced efficiency (with a factor of ~2) when the substrate was relatively hydrophilic. It was established that the polyelectrolyte deposition could be exploited to effectively tune the substrate surface hydrophobicity, thereby providing more degrees of freedom for the optimization of the TFC membranes fabrication.

## 1. Introduction

Thin film composite (TFC) membranes are essential for various membrane-based applications, such as seawater desalination, ultrapure water production and advanced wastewater treatment [1,2], which play an important role in the sustainable development of the economy and society. The superior performance of TFC membranes can be attributed to the ultrathin selective layer that is mainly formed via interfacial polymerization (IP). Numerous endeavors have been devoted to optimizing the IP process to improve the permeability and selectivity of a TFC membrane [3,4]. Despite the increase in studies focusing on fabricating TFC membranes [4,5], limited knowledge is available about the mechanisms accounting for the formation of an IP film, which are of critical importance to the improvement of the performance of TFC membranes.

Most prior studies investigated the selective layer (i.e., the polyamide layer) and the support layer (i.e., the substrate) of a TFC membrane separately [6,7], and their focus was usually on the optimization of the selective layer. There are a limited number of studies that explore the effects of the substrate on the formation of a polyamide (PA) layer. It has been recognized that both the surface morphology (especially the pore-size distribution) and surface hydrophilicity of the substrate are two of the most important factors affecting the formation of a PA layer [8,9]. Singh et al. [10] explored the effects of substrate structures on the formation of the PA-layer by employing two polysulfone substrates with varied pore-size distributions. It was revealed that increasing the pore size could lead to a relatively thin PA layer with a higher permeability. Similar observations were obtained by Ghosh and Hoek in a later study [8]. In addition to varying the pore size, the effects of the morphology were highlighted in the work by ElSherbiny et al. [11], where a novel polyethersulfone substrate with isotropic pores (~100 nm) was developed by combining the vapor- and non-solvent-induced phase separation; this novel substrate yielded a TFC membrane with a higher water permeability compared with those fabricated using commercial substrates, while maintaining the salt rejection. Recently, Mohammadifakhr et al. [12] employed a single-step process to incorporate an intermediate layer formed by oppositely charged polyelectrolytes on a hollow fiber substrate; the membrane performance was significantly enhanced after IP. In comparison with the studies on the effects of substrate structures, there are substantially different opinions in the open literature interpreting the role of the hydrophilicity of the substrate in the IP process.

The preference of a hydrophobic substrate for IP was supported by prior studies in different ways. For example, Ghosh and Hoek [8] proposed a conceptual model suggesting that a relatively hydrophobic substrate would result in a highly permeable TFC membrane. Specifically, it was hypothesized that enhancing the hydrophilicity of the substrate could impede the diffusion of m-phenylenediamine (MPD) in the aqueous phase while promoting the diffusion of trimesoyl chloride (TMC) in the organic phase into the substrate pores, thus forming a thicker PA layer during IP. In contrast, the work by Alsvik et al. [13] highlighted the delamination phenomenon of the PA layer formed on a hydrophilic substrate of hydrolyzed cellulose acetate (CA). In order to improve the adhesion between the PA layer and the hydrophilic substrate, they proposed to precoat the substrate with a TMC-containing solution, taking advantage of the hydroxyl groups on the CA network; this TMC-pre-coated substrate would allow the covalent bonding with the PA layer and the hydrophilic substrate. The delamination of the active layer was also observed by Zhang et al. [14] when using a series of polysulfone (PSf) substrates for the fabrication of the TFC membranes via IP, though the underlying mechanism remained unclear.

On the contrary, there are prior studies indicating that increasing the hydrophilicity of the substrate could not only enable the membrane-supported IP but also improve the performance of the resulting TFC membrane. For example, Kim et al. [15] demonstrated that a plasma treatment would allow polypropylene (PP) and PSf membranes to be used as the substrate for IP, owing to the increase in the hydrophilicity. A similar method was adopted by Kim et al. [16] to successfully fabricate a TFC membrane via IP using a hydrophobic polyvinylidene fluoride (PVDF) substrate. In addition to the plasma treatment, it was reported that the hydrophilicity of the substrate could be enhanced by surface coating and incorporating hydrophilic additives into the dope solutions, so as to favor the formation of a PA layer via membrane-supported IP, and to improve the TFC membrane performance. For instance, Jimenez-Solomon et al. [17] successfully fabricated TFC membranes via IP using crosslinked polyimide and poly(ether ether ketone) substrates, whose hydrophilicity was increased by simply dipping the substrates into a solution of polyethylene glycol before the IP; Ding et al. [18] examined the effects of the substrate hydrophilicity on the performance of the TFC membranes fabricated via IP by varying the amount of polyvinylpyrrolidone mixed into the PSf dope solutions, which were used to prepare the substrates via phase inversion.

Despite the discrepancy between the studies on the role of the substrate hydrophilicity in the formation of a PA layer via membrane-supported IP, it has been widely accepted that a hydrophilic substrate would substantially improve the performance of a TFC membrane employed in an osmotically driven process, owing to the mitigation of the internal concentration polymerization (ICP) and fouling [19]. Although the potential of the application of osmotically driven membrane processes to desalination remains controversial [20,21], great promise has been shown in their applications for pharmaceutical production [22], the food and beverage industry [23], emergency relief [24], wastewater treatment [25], and energy generation [26]. Therefore, it is of great significance to develop TFC membranes with a hydrophilic substrate for various osmotically driven membrane processes.

A limited number of studies reported the development of an effective method for synthesizing TFC membranes with a hydrophilic substrate. In addition to the method based on the TMC precoating by Alsvik et al. [13], Choi et al. [27] successfully fabricated a nanoscale-controlled PA layer with a typical thickness ~15 nm by depositing the complementary monomers (i.e., MPD and TMC) alternatingly on a polyelectrolyte-modified hydrophilic polyacrylonitrile (PAN) substrate; Park et al. [28] employed toluene/xylene as the solvent for the organic phase, and synthesized TFC membranes with higher performance for reverse osmosis (RO) using a hydrophilic PAN membrane as the substrate; the method developed by Park et al. [28] was further optimized by Kwon et al. [29], and the synthesized TFC membranes were examined in a forward osmosis (FO) process. However, most of these studies ignored the effects of the substrate hydrophilicity on the formation of the PA layer.

Instead of targeting the fabrication of TFC membrane with superior performance, the focus of the current study was on the role of the substrate hydrophilicity in a membrane-supported IP process. In line with this idea, the formation of a PA layer via membrane-supported IP was investigated while varying the hydrophilicity of the substrate. In particular, it was proposed to tune the surface hydrophilicity of the substrate via the deposition of various polyelectrolytes, which have been extensively explored for the Layer-by-Layer (LbL) assembly [30,31]. The relative importance of the enhanced surface hydrophobicity in the IP-film formation was analyzed in terms of a series of characterization experiments. It was confirmed that the polyelectrolyte deposition could increase the surface hydrophobicity of a hydrolyzed PAN substrate, and could thereby favor the formation of an IP layer with better integrity (i.e., minimized delamination). The performance of the fabricated TFC membranes with the hydrolyzed PAN substrate was evaluated in an FO process in order to verify the mitigation of the ICP by the hydrophilic substrate.

## 2. Experimental Section

### 2.1. Interfacial Polymerization on Substrates with Varied Hydrophilicity

The substrates employed for the membrane-supported IP in this study were all fabricated using polyacrylonitrile (PAN) via phase inversion, the various applications of which were reported in our previous publications [32,33]. Specifically, the dope solution was prepared by dissolving polymer particles of PAN (weight-averaged molecular weight M_w_ ~150,000 g/mol, Sigma-Aldrich, St. Louis, MO, USA) in N,N-dimethylformamide (DMF, ≥99.8%, Alfa Aesar, Shanghai, China) with a predetermined amount of lithium chloride (LiCl, ≥99%, Sigma-Aldrich, St. Louis, MO, USA) as the pore former [32,33]. The mass ratio of the PAN, LiCl, and DMF in the dope solution was 18:2:80. The dope solution was mixed using a magnetic stirrer (IKA, C-MAG HS7 Control, Staufen, Germany) at a temperature of ~60 °C until it became transparent and homogeneous. The cooled and degassed dope solution was cast on a smooth, flat glass plate using a film applicator (ANMT, AT-TB-2100, Jinan, China) when setting the gate height of the casting knife (Zehntner GmbH Testing Instruments, ZUA 2000, Sissach, Switzerland) at a value of 150 μm. The film-coated glass plate was immediately immersed into a coagulation bath of tap water at room temperature (~20 °C) in order to initiate the nonsolvent-induced phase inversion. The precipitated PAN films were rinsed under running tap water for 24 h in order to remove the residual chemicals, followed by a rinse of Milli-Q water (Milli-Q^®^ Direct 8 Water Purification System, Molsheim, France). The unmodified PAN films were denoted as ‘PAN-O’, and were used as the substrates in the control tests.

The hydrophilicity of the PAN-O films was first changed by implementing an alkaline treatment, whereby the nitrile groups would be converted into carboxyl groups [34]. Specifically, the PAN-O films were soaked into a 1.5 M NaOH (≥96%, Fuchen Chemical, Shanghai, China) solution at 45 °C for 90 min [32,33]. The resulting PAN substrates were designated as ‘PAN-A’. It was revealed in prior studies [35] that the pore size of the PAN substrate could be decreased during the alkaline treatment, owing to the high temperature (i.e., the decrease in the free volume owing to the rearrangement of the molecular chains). In order to isolate the effects resulting from the pore-size change, a comparative study was carried out by applying a heat treatment to the PAN-O films. Specifically, the PAN-O substrates were immersed in a bath of Milli-Q water while the temperature and duration were kept at the same values for the alkaline treatment (i.e., 45 °C for 90 min). The resulting PAN substrates were denoted as ‘PAN-H’. All of the PAN substrates were stored in Milli-Q water before the characterization and IP experiments.

The membrane-supported IP was performed by impregnating the PAN substrates with an aqueous solution containing 2.0 wt.% *m*-phenylenediamine (MPD, ≥99%, Sigma-Aldrich, St. Louis, MO, USA) and exposing the impregnated PAN substrates to an organic solution containing 0.1 w/v.% 1,3,5-benzenetricarbonyl trichloride (TMC, ≥99%, Sigma-Aldrich, St. Louis, MO, USA). In particular, the solvent of the TMC-containing organic solution was hexane (≥98%, Shanghai Aladdin Biochemical Technology Co. Ltd., Shanghai, China); the duration for the soaking of the PAN substrates in the MPD-containing aqueous solution was approximately 5 min, and the excessive MPD solution was removed using an air knife of compressed N_2_ [1]; the duration for the exposure of the impregnated PAN substrates to the TMC-containing organic solution was approximately 1 min, and the residual TMC was removed by a rinse of pure hexane. The fabricated TFC membranes were stored in Milli-Q water before the characterization. All of the PAN substrates and the TFC membranes are summarized in Table 1.

### 2.2. Tuning the Hydrophilicity of Substrates via Polyelectrolyte Deposition

The PAN-A substrates enabled the surface modification via the deposition of polycations, owing to the negatively charged surface after the alkaline treatment (i.e., the formation of carboxyl groups). It was expected that the deposition of various polycations would significantly alter the hydrophilicity of the PAN-A substrates [36]. In particular, three polycations were employed in order to modify the PAN-A substrates, including poly(allylamine hydrochloride) (PAH, M_w_ ~120,000 to 200,000 g/mol, ≥99%, Alfa Aesar, Shanghai, China), polyethyleneimine (PEI, M_w_ ~750,000 g/mol, 50%, Sigma-Aldrich, St. Louis, MO, USA), and poly(dimethyl diallyl ammonium chloride) (PDADMAC, M_w_ ~200,000 to 350,000 g/mol, 20%, Sigma-Aldrich, St. Louis, MO, USA). The deposition of the polycations on the PAN-A substrates was implemented in a way that was similar to that employed by the Layer-by-Layer assembly using polyelectrolytes [32,33]. Specifically, 1 g of the polycation was dissolved in 1 L Milli-Q water containing 0.5 M sodium chloride (NaCl, ≥99.5%, Xilong Scientific, Guangzhou, China); the existence of the NaCl would favor the assembly of the polyelectrolyte on the surface of the substrate [37]. The PAN-A substrate was placed in a stainless steel plate with the dense side facing up. A predetermined amount of the polycation solution was gently poured into the plates to contact with the surface of the PAN-A substrate. The duration of the exposure of the PAN-A substrate to the polycation solution was 20 min, and the deposition was followed by a 10 min rinse of Milli-Q water to remove the excess polyelectrolyte on the substrate. The polyelectrolyte-modified substrates were stored in Milli-Q water before the IP experiments, and were denominated as ‘XXX-m’, where XXX indicates the specific polyelectrolyte used for the substrate modification. All of the polyelectrolyte-modified substrates are summarized in Table 1.

### 2.3. Membrane Characterization

The hydrophilicity of the PAN substrates was characterized in terms of the contact angle measurement. All of the PAN substrates were dried using a freeze dryer (Ningbo Scientz Biotechnology Co. Ltd., Scientz-12N, Ningbo, China) before the contact angle measurement, and the sessile drop method was employed using a Drop Shape Analyzer (KRÜSS GmbH, DSA25, Hamburg, Germany). In particular, the contact angle was estimated by invoking the Young–Laplace algorithm [38], and the measurement was repeated (at least nine times using three independently prepared membrane samples) to obtain an averaged value for a more accurate analysis.

It has been revealed in prior studies [39] that the surface morphology could play an important role in the surface wetting. Therefore, both scanning electron microscopy (SEM) and atomic force microscopy (AFM) were exploited in order to characterize the surface of the PAN substrates. In particular, the SEM characterization was performed by precoating the membrane samples with platinum in a vacuum electric sputter coater (Quorum, Q150TES, East Grinstead, UK) and then observing the precoated samples on an SEM system (ZEISS, Merlin^TM^, Oberkochen, Germany). The surface roughness of the PAN substrates was analyzed using an AFM system (Asylum Research, MFP-3D^TM^ Stand Alone, Santa Barbara, CA, USA) associated with Asylum Research. The roughness was quantified in terms of the root mean square (RMS) values that were obtained from the measurements of three independently prepared membrane samples.

The TFC membranes were characterized using an osmotically driven process, whereby the efficiency of the FO was estimated to assess the effect of the substrate on the ICP. Specifically, the pure water permeability (*A*) of the membrane was measured using a high pressure crossflow-filtration setup (Fumei Filter & Membrane Technology, FlowMem0021-HP, Xiamen, China) and calculated in terms of the flux-driving force relation as the following equation [40]:

(1) $$A=\frac{J_{V}}{\Delta P-\Delta \pi }$$

where *J_V_* is the water flux obtained by weighting the permeate [32,33], Δ*P* is the trans-membrane pressure (the Δ*P* was 5 bar for all the tests in the current study), and Δ*π* is the osmotic pressure difference across the membrane that can be ignored for the measurement using pure water. The osmotically driven process was performed by employing a 2 M NaCl solution as the draw solution (DS), and the water flux was measured by weighing the feed solution (i.e., pure water). The efficiency of the FO (*η_FO_*) was then calculated in terms of the following equation:

(2) $$\eta_{FO}=\frac{J_{V}}{A\cdot \Delta \pi }\times 100\%$$

In addition, the salt rejection (*R*) of the TFC membrane was determined in terms of the conductivity measurement, with a 20 mM NaCl solution as the feed. The conductivity was measured using a conductivity meter (Mettler Toledo, FE38, Greifensee, Switzerland), and the rejection was estimated by the following equation [41]:

(3) $$R=\left(1-\frac{C_{p}}{C_{f}}\right)\times 100\%$$

where *c_p_* and *c_f_* are the concentrations of NaCl in the permeate and feed solutions, respectively, which can be obtained by the linear relationship between the conductivity and the salt concentration [42]. All of the measurements were repeated at least three times to obtain the averaged values for a more accurate analysis.

## 3. Results and Discussion

### 3.1. Effects of the Substrate Hydrophilicity on the IP-Film Formation

In order to vary the hydrophilicity of the substrate while maintaining the geometric characteristics, the PAN substrate was hydrolyzed in a heated NaOH solution in order to introduce carboxyl groups onto the membrane surface; the effect of the heating on the PAN substrate was accounted for by comparing the results with those obtained by soaking the PAN membrane in heated pure water. The contact-angle measurement was implemented for the PAN-O (i.e., the original PAN membrane), PAN-H (i.e., the PAN membrane soaked in heated water), and PAN-A (i.e., the PAN membrane pretreated in a heated NaOH solution) substrates to reveal the variation in the hydrophilicity of the membrane surface.

**Figure 1:**
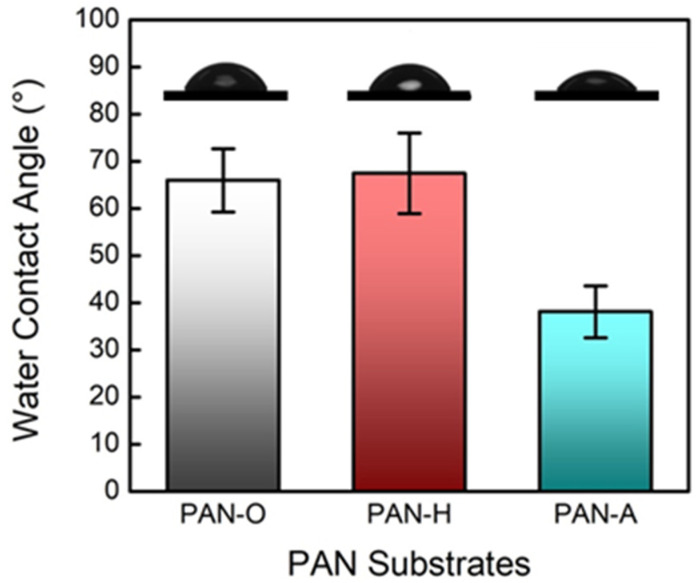
Comparison of the contact angles measured on the surface of the original PAN substrate and the PAN substrates with the heat treatment and alkaline treatment. The error bars correspond to the standard deviations of the characterization results (nine measurements using three membrane samples).

The contact-angle measurement results (including the averaged values of the measured contact angles and the representative sessile drop images) are shown and compared in Figure 1. The contact angle of approximately 67° for the PAN-O membrane confirms that PAN is relatively hydrophilic, in comparison with the other support materials (e.g., PSf) for the fabrication of TFC membranes via IP [43]. The negligible difference in the contact angle between the PAN-O and PAN-H membranes indicates that the heat treatment with a temperature of 45 °C had little impact on the hydrophilicity of the PAN substrate. In contrast, the introduction of the carboxyl groups by the alkaline treatment markedly increased the hydrophilicity of the PAN substrate, as indicated by the decrease in the contact angle for the PAN-A membrane in Figure 1.

**Figure 2:**
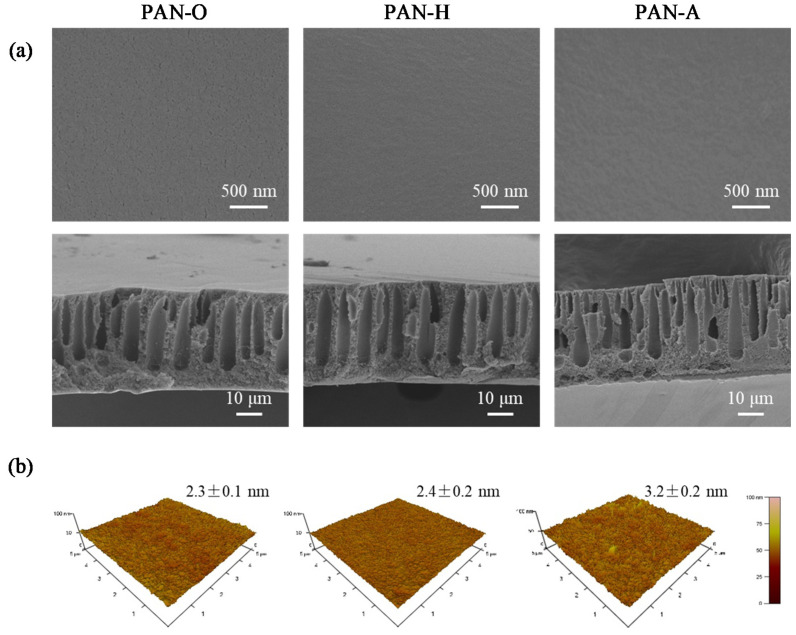
Comparison of the geometrical characteristics of the original PAN substrate and the PAN substrates with the heat treatment and alkaline treatment via (**a**) SEM and (**b**) AFM. The SEM images demonstrate both the top and cross-sectional views of the PAN substrates; the AFM images are displayed with the associated roughness values in terms of the RMS.

The effects of the heat and alkaline treatments on the surface morphology of the PAN substrates were examined using both SEM and AFM; the characterization results are shown in Figure 2a,b, respectively. The SEM images in the upper panel of Figure 2a exhibit the dense side of the three PAN substrates. It can be observed that there exist nano-sized pores on the surface of both the PAN-O and PAN-H substrates, though the sizes of the pores on the surface of the PAN-H substrate are substantially smaller than those on the surface of the PAN-O substrate. On the other hand, there are no discernable pores in the SEM image for the surface of the PAN-A substrate. All of these observations indicate that the PAN polymer network was shrunk, owing to the stress relaxation of PAN polymer in the water bath at an elevated temperature of 45 °C [44]; the swelling of the PAN polymer network resulting from the alkaline treatment further reduced the pore size [45,46], which is probably due to the synergic effect of the alkaline treatment and the elevated temperature. The enlarged SEM images of the original PAN substrate and the PAN substrates with the heat treatment and alkaline treatment are exhibited in Figure S1.

Although the pore shrinkage was significant on the dense side of the post-treated PAN substrates, the heat and alkaline treatments could have marginal effects on the figure-like macrovoids beneath the dense skin. This hypothesis can be visually validated by comparing the cross-sectional SEM images of the PAN-O, PAN-H, and PAN-A substrates shown in the lower panel of Figure 2a. It is reasonable to assume that the pore shrinkage of the sponge-like substructures would contribute less to the IP process if the macrovoids were more easily wetted by the MPD-containing solution.

The surface of the dense side of the PAN substrates was further characterized by AFM to evaluate the changes in the roughness, which could result from the post-treatments. The representative AFM images associated with the corresponding values of the roughness are displayed in Figure 2b. It is interesting to note that, despite the difference in the pore size, all of the PAN substrates have similar values of roughness in terms of the root mean square (RMS); the RMS roughness values for the PAN-O and PAN-H substrates are nearly the same (approximately 2.4 ± 0.2 nm), while that of the PAN-A substrate is increased to 3.2 ± 0.2 nm. The AFM characterization results provide quantitative evidence to support the assumption that the geometrical changes resulting from the post-treatments could be ignored when analyzing the effects of the substrate on the IP process; this assumption is of critical importance in the current study that highlights the role of the surface hydrophilicity.

**Figure 3:**
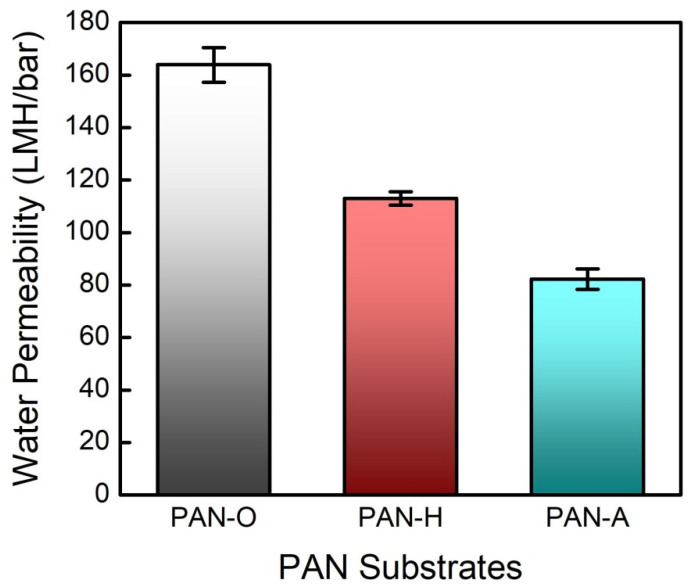
Evaluating the original PAN substrate and the PAN substrates with the heat treatment and alkaline treatment in terms of their water permeabilities. The flux–pressure relationship was determined with a hydraulic pressure of 5 bar; the error bars correspond to the standard deviations of three independent measurements.

The water permeabilities of the PAN substrates were evaluated in terms of the flux-transmembrane pressure (TMP) relationship, and the evaluation results are compared in Figure 3. It is evident that both the heat and alkaline treatments substantially increased the hydraulic resistance of the substrate, i.e., they gave a lower value of the water permeability in comparison with that for the PAN-O substrate (163.9 ± 6.6 LMH/bar). In particularly, the hydrolysis in the NaOH solution resulted in an even lower value of the water permeability (82.2 ± 3.9 LMH/bar) when compared with that for the PAN-H substrate (113.0 ± 2.6 LMH/bar). These observations are consistent with the changes in the surface pore sizes on the dense side, as revealed by the SEM images in Figure 2a.

**Figure 4:**
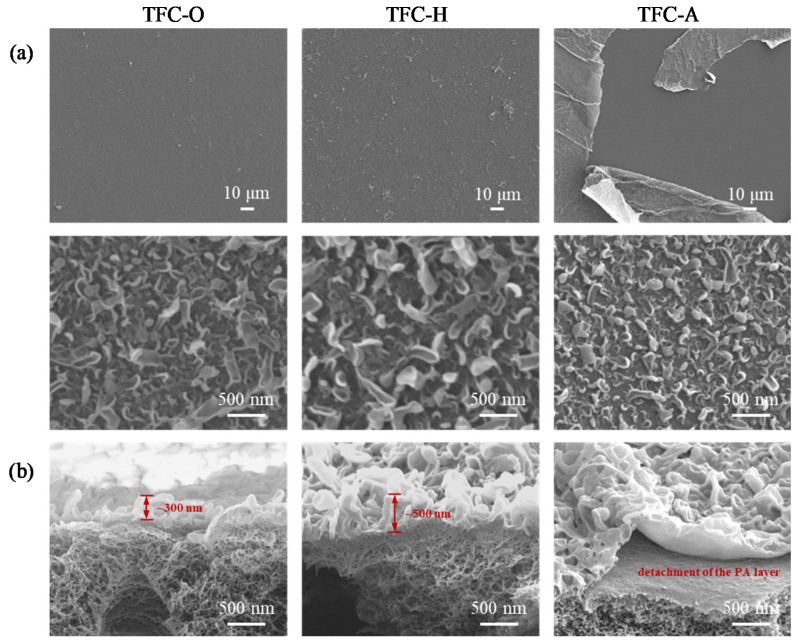
Comparison of the IP layers formed on the original PAN substrate and the PAN substrates with the heat treatment and alkaline treatment, in terms of the SEM images showing (**a**) the top views with varied magnifications and (**b**) the cross-sectional views. The IP was implemented by contacting the PAN substrate (impregnated with the aqueous solution of 2.0 wt% MPD) with the organic solution (0.1 w/v.% TMC dissolved in hexane).

In order to elucidate the effects of the substrate hydrophilicity on the formation of a PA layer, TFC membranes were fabricated using the PAN-O, PAN-H, and PAN-A substrates via IP (denoted by TFC-O, TFC-H, and TFC-A, respectively). The TFC membranes were characterized by SEM and the SEM images of the top view with varied scales and cross-sectional views, which are demonstrated in Figure 4a,b, respectively. It is worthwhile to note that the delamination of the TFC-A membrane occurred immediately after the IP process, without performing any filtration tests. The upper panel of Figure 4a shows the top views at a lower magnification, such that a larger area can be covered for the observation. It evidently indicates that the IP film was not firmly ‘locked’ by the surface of the PAN-A substrate, and it irregularly split into smaller pieces with curved or folded edges. In contrast, both the PAN-O and PAN-H substrates yielded a relatively uniform and ‘undamaged’ IP layer, whose typical ridge and valley substructures [4,47] are resolved by zooming in on the surface of the IP layer (the lower panel of Figure 4a). The SEM image with a higher magnification also reveals similar substructures of the IP layer peeling off from the PAN-A substrate.

The cross-sectional views in Figure 4b provide further evidence for the delamination of the TFC membrane fabricated using the PAN-A substrate. These observations are consistent with the work by Alsvik et al. [13], arguing that the hydrophilicity of the substrate could result in unfavorable interactions between the generated IP layer and the substrate. It is also interesting to note that the PAN-H substrate yielded an IP layer with thicker substructures (approximately 500 nm) in comparison with those (approximately 300 nm) resulting from the PAN-O substrate. The shrinkage of the surface pores might increase the resistance for the diffusion of the MPD in the aqueous phase, and might thereby favor the development of the polymerization toward the aqueous phase, which is similar to the case of a relatively low concentration of MPD (i.e., limited diffusion on the aqueous side) in the work by Chai and Krantz [48]. When the evolution of the coupled diffusion and reaction processes was spatially dominated by the porous surface of the substrate, it was reasonable to expect that the polymerization should be perturbed in a destabilizing way in order to give rise to more periodic patterns, such as the extended ‘leaves’ [47].

**Figure 5:**
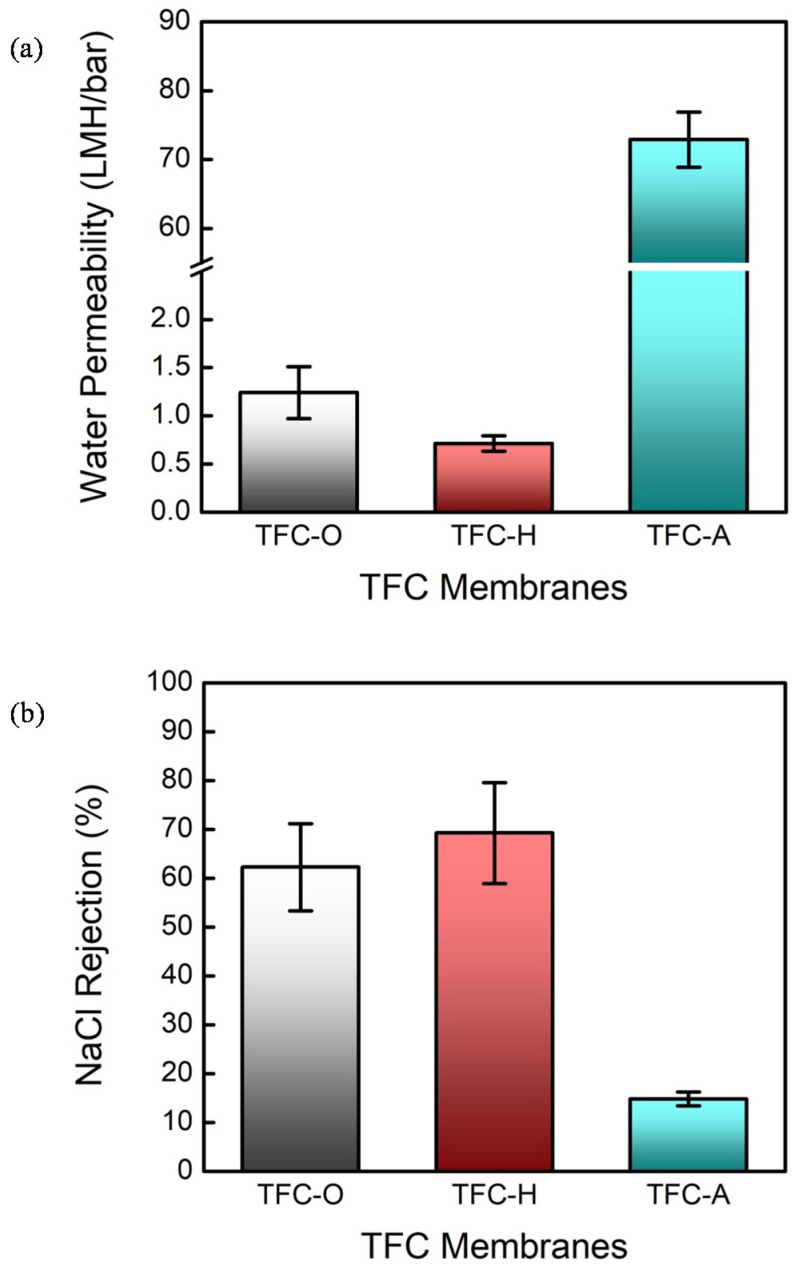
Evaluating the TFC membranes with the original PAN substrate and the PAN substrates with the heat treatment and alkaline treatment in terms of (**a**) their water permeabilities and (**b**) their rejections of sodium chloride. The flux–pressure relationship was determined with a hydraulic pressure of 5 bar; the salt rejection was measured using an aqueous feed solution of 20 mM NaCl; the error bars correspond to the standard deviations of the three independent measurements.

Another way of verifying the delamination of the TFC membrane is to measure the water permeability and salt rejection of all of the TFC membranes fabricated using the different PAN substrates. The experimental results for the water permeability and salt rejection of NaCl are displayed in Figure 5a,b, respectively. The integrity of the IP layers formed on the PAN-O and PAN-H substrates is confirmed by comparing the values of the water permeability for the TFC membranes with different PAN substrates; that is, the water permeabilities for the TFC-O and TFC-H are decreased to a value comparable with commercially available TFC membranes [4], whereas the one for the TFC-A (~72.9 LMH/bar) is almost equal to that for the substrate, as shown in Figure 3. On the other hand, it is not surprising to note that the salt rejection of NaCl for the TFC membrane with the PAN-A substrate (14.8 ± 1.4%) is much lower than those for the TFC membranes with the PAN-O and PAN-H substrates (62.3 ± 8.9% and 69.3 ± 10.4%, respectively), since the active layer (i.e., the IP layer) peeled off on the surface of the PAN-A substrate. Moreover, the TFC-H has a relatively low water permeability compared with that for the TFC-O substrate, whereas the corresponding salt rejection of NaCl is slightly increased. This observation is consistent with the SEM characterization result showing an IP layer with relatively thicker substructures for the TFC membrane with the PAN-H substrate, thereby offering additional evidence for the integrity of the TFC-O and TFC-H.

### 3.2. Effects of the Polyelectrolyte Deposition on the IP-Film Formation

It has been demonstrated that increasing the hydrophilicity of the substrate could result in the delamination of the TFC membrane fabricated via IP; that is, the hydrophilic substrate surface would not favor the adhesion of the IP layer, or the formation of self-locking structures between the IP layer and the substrate. However, it was tempting to employ a hydrophilic substrate for fabricating a TFC membrane via IP owing to the potential advantages for improving the efficiency during an FO process. Therefore, the polyelectrolyte deposition was exploited in order to modify the wettability of the substrate surface.

**Figure 6:**
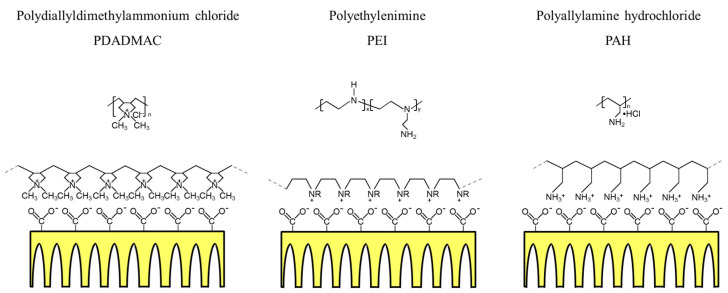
Schematics demonstrating the deposition of various polycations (including PDADMAC, PEI, and PAH) onto the PAN substrate with the alkaline treatment.

Three polycations, including PDADMAC, PEI, and PAH, were employed in the current study to tune the surface of the PAN-A substrate, which was negatively charged, owing to the existence of the carboxyl groups. The deposition of the polycations onto the PAN-A substrate is schematically shown in Figure 6, ignoring the conformational effects of the polymeric chains. It was expected that the hydrophilicity of the PAN-A could be decreased by the deposition of the polycations with the nonpolar segments exposed to the liquid phase, though prior studies [49,50] indicated that the degree of hydrophilicity should be dependent on the various conditions (e.g., ionic strength and pH value) employed by the polyelectrolyte deposition.

**Figure 7:**
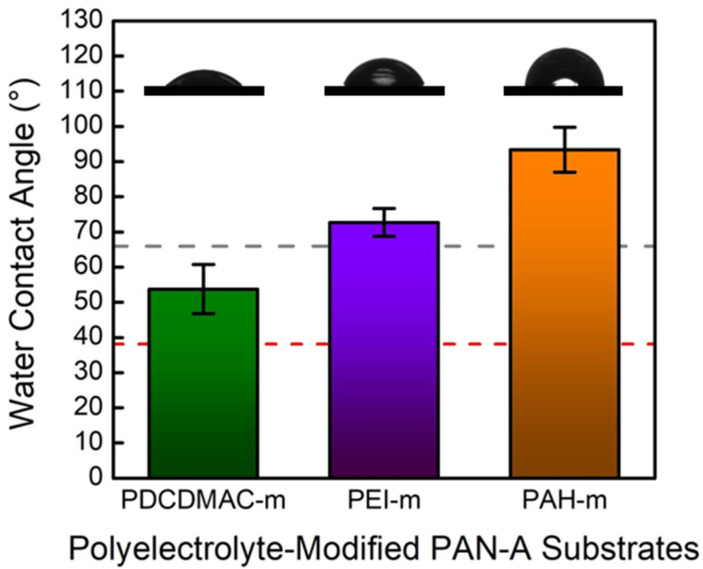
Comparison of the contact angles measured on the surface of the PAN-A substrates modified with the deposition of PDADMAC, PEI, and PAH. The surface modification was implemented by exposing the dense side of the PAN-A substrates to an aqueous solution containing 1 g of the polyelectrolyte and 0.5 M NaCl for 20 min. The gray dashed line marks the average value of the contact angle measured on the surface of the original PAN substrate; the red dashed line marks the average value of the contact angle measured on the surface of the PAN substrate with the alkaline treatment; the error bars correspond to the standard deviations of the characterization results (nine measurements using three membrane samples).

The contact-angle measurement results (including the averaged values of the measured contact angles and the representative sessile drop images) for the polyelectrolyte-modified PAN-A substrates are shown and compared in Figure 7. In particular, the red dashed line denotes the average contact angle for the PAN-A substrate, while the gray dashed line is for the PAN-O substrate. It clearly indicates that the deposition of all of the polycations significantly decreased the degree of the surface hydrophilicity (i.e., increased the contact angle) for the PAN-A substrate. The PAN-A substrate modified by PDCDMAC yielded the smallest contact angle (52.1 ± 6.5°), which was greater than that for the unmodified PAN-A substrate (38.1 ± 5.5°). On the other hand, the degrees of the surface hydrophobicity for the PAN-A substrates modified with PEI and PAH were even higher than that for the PAN-O substrate (i.e., larger contact angles). In particular, the PAN-A substrate modified with PAH yielded the largest contact angle greater than 90°, indicating a hydrophobic surface in a typical sense [51]. Note that these polyelectrolytes could introduce additional functional groups (e.g., primary and secondary amine groups) to react with the acyl chloride. However, the substantial increase in the surface hydrophobicity indicates that most of the nonpolar segments should be exposed to the liquid phase while ‘preventing’ the polar groups (i.e., the amine groups) from meeting with the TMC in the organic phase. It is reasonable to assume that the effects of the functional groups introduced by the polyelectrolytes could be ignored, in order to focus on the variation in the hydrophobicity.

**Figure 8:**
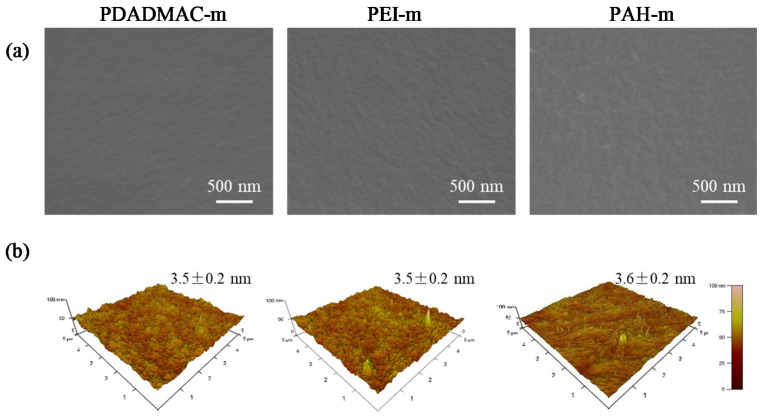
Comparison of the geometrical characteristics of the PAN-A substrates modified with the deposition of PDADMAC, PEI, and PAH via (**a**) SEM and (**b**) AFM. The SEM images demonstrate both the top views of the modified PAN-A substrates; the AFM images are displayed along with the associated roughness values in terms of the RMS.

The PAN-A substrates modified with various polycations were characterized by SEM and AFM in a similar way to support the assumption that the deposition of the polycations substantially varied the wettability while having a marginal impact on the geometrical characteristics of the surface of the PAN-A substrate. The SEM and AFM characterization results are shown and compared in Figure 8a,b, respectively. In terms of the SEM images, the polyelectrolyte deposition further ‘removed’ the pore structures, which are discernable in the SEM image for the PAN-O substrate in Figure 2a. All of the polyelectrolyte-modified PAN-A substrates yielded an RMS value of roughness around 3.5 nm. Despite the marginal difference in the geometrical characteristics, it is interesting to note that the PAN-A substrate modified with PAH gave rise to a slightly high value of the surface roughness; this is consistent with the observation in prior studies [50] that relatively high heterogeneity could result from the deposition of PAH.

**Figure 9:**
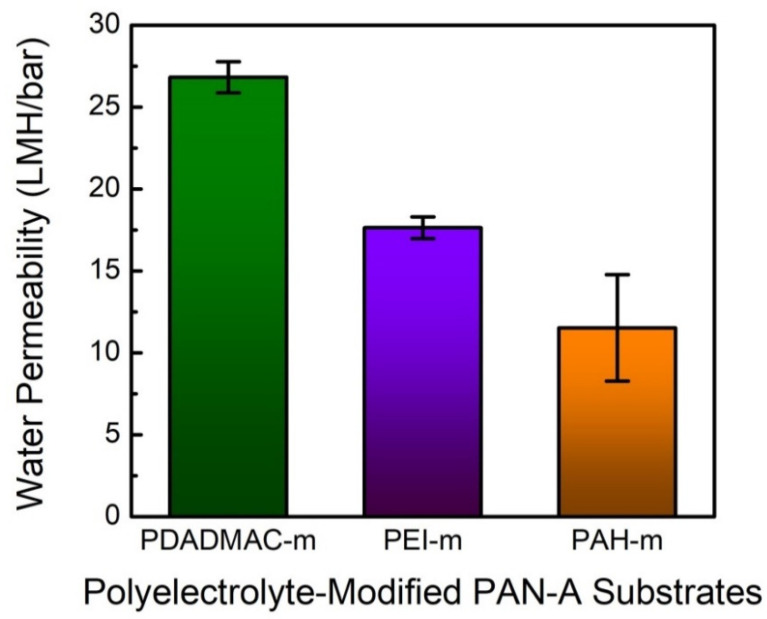
Evaluating the PAN substrates modified with the deposition of PDADMAC, PEI, and PAH in terms of their water permeabilities. The flux–pressure relationship was determined with a hydraulic pressure of 5 bar; the error bars correspond to the standard deviations of three independent measurements.

The water permeabilities of the polyelectrolyte-modified PAN-A substrates are displayed and compared in Figure 9. It is affirmed that the deposition of the polycations significantly increased the hydraulic resistance of the PAN-A substrate, even though the deposited layer should have a negligible thickness of a few nanometers [31]. Moreover, the variation in the water permeability is consistent with the varying wettability, characterized by their contact angles in Figure 7. That is, the relatively hydrophilic surface of the PAN-A substrate modified with PDADMAC yielded the highest water permeability, whereas the lowest water permeability corresponded to the hydrophobic surface resulting from the deposition of the PAH.

**Figure 10:**
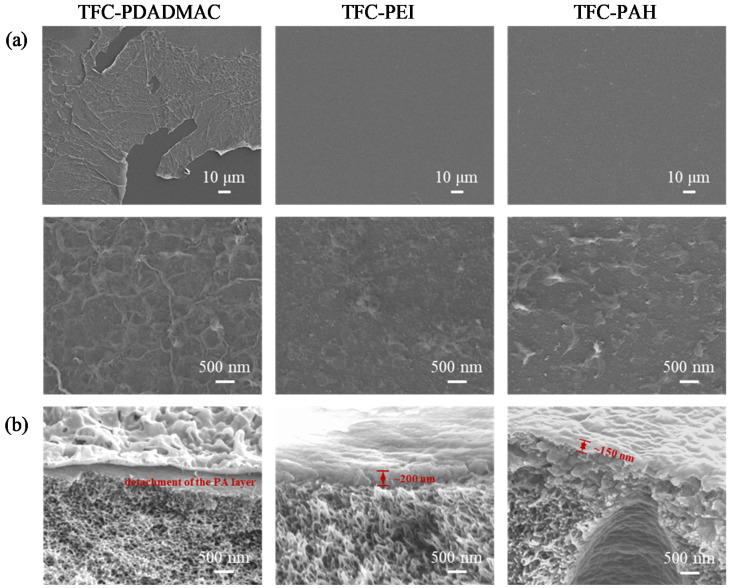
Comparison of the IP layers formed on the PAN-A substrates modified with the deposition of PDADMAC, PEI, and PAH in terms of the SEM images, showing (**a**) the top views with varied magnifications and (**b**) the cross-sectional views. The IP was implemented by contacting the PAN-A substrate (impregnated by the aqueous solution of 2.0 wt.% MPD) with the organic solution (0.1 w/v.% TMC dissolved in hexane).

A series of TFC membranes were fabricated using the polyelectrolyte-modified PAN-A substrates (correspondingly denoted by TFC-PDADMAC, TFC-PEI, and TFC-PAH). The TFC membranes could be considered as three-layer membranes, where the ultrathin polyelectrolyte layer serves as an intermediate layer between the PAN-A substrate and the PA layer. The samples of the TFC membranes were characterized by SEM in order to generate the top views with varied magnifications and cross-sectional views, as presented in Figure 10a,b. It is evident, in terms of the top views at a larger magnification (the upper panel in Figure 10a), that delamination occurred during the IP for the fabrication of the TFC-A and TFC-PDADMAC. This observation is consistent with the hypothesis that a surface more hydrophilic than that of the PAN-O substrate could be unfavorable for ‘locking’ the generated IP layer; the water wettability of the surface modified with PDADMAC was slightly higher than that of the PAN-O substrate, though the deposition of PDADMAC somehow increased the hydrophobicity of the surface of the PAN-A substrate, as revealed by the contact-angle characterization in Figure 7.

Despite the occurrence of delamination, all of the IP layers generated on the polyelectrolyte-modified PAN-A substrates were characteristic of relatively dense and smooth substructures in terms of the top views at a larger magnification (lower panel of the SEM images in Figure 10a). A possible explanation is that the relatively hydrophobic surface could favor the diffusion of TMC in the organic phase toward the interface of polymerization, and could thereby enhance the degree of crosslinking [52], which might limit the growth of heterogeneous substructures. On the other hand, the cross-sectional views in Figure 10b indicate that relatively thin IP layers (around 200 nm) were formed on the surface of the PAN-A substrates modified with PEI and PAH. This observation is consistent with the hypothesis that the film formation should be dominated by the crosslinking that yielded a relatively dense network and accelerated the IP process toward the self-limiting regime [53]. In addition, the cross-sectional view for the TFC-A and TFC-PDADMAC also verifies the detachment of the IP layer from the substrate.

**Figure 11:**
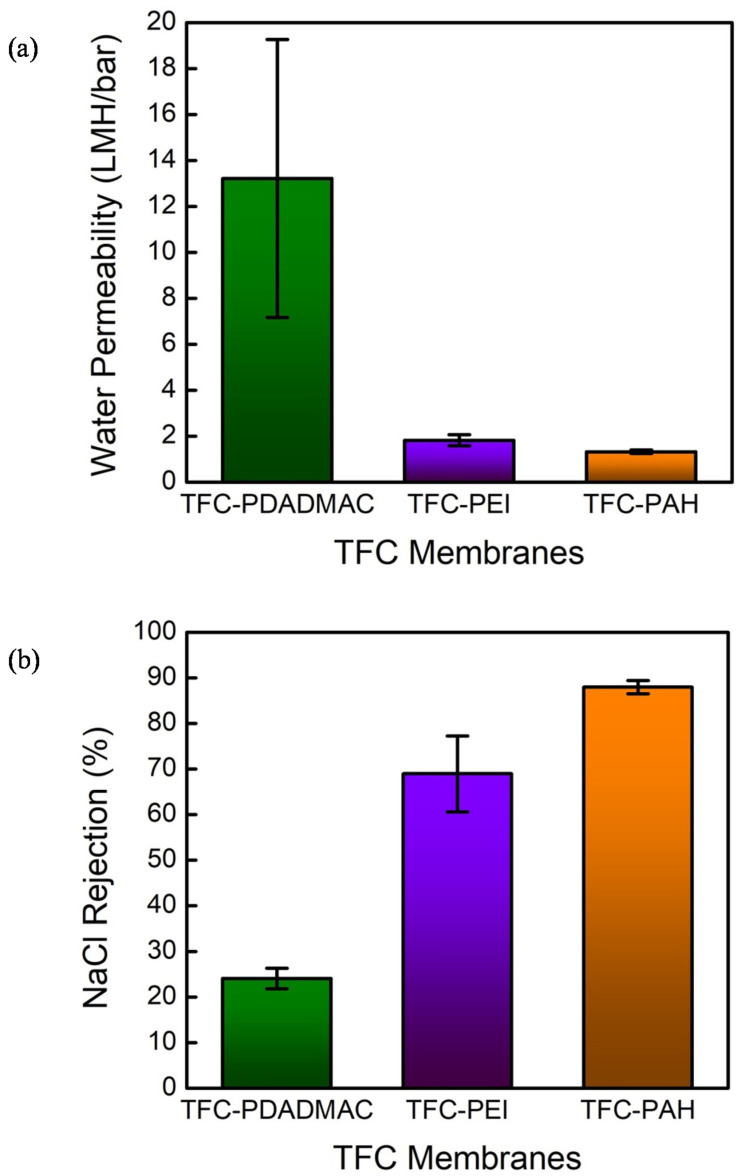
Evaluating the TFC membranes with the PAN-A substrates modified with the deposition of PDADMAC, PEI, and PAH in terms of (**a**) their water permeabilities and (**b**) their rejections of sodium chloride. The flux–pressure relationship was determined with a hydraulic pressure of 5 bar; the salt rejection was measured using an aqueous feed solution 20 mM NaCl; the error bars correspond to the standard deviations of the three independent measurements.

The integrity of the TFC-PEI and TFC-PAH is confirmed by the characterization results of the water permeability and NaCl rejection, which are displayed in Figure 11a,b, respectively. Although the TFC-PAH yielded a relatively low value of water permeability (~1.3 LMH/bar) in comparison with that for the TFC-PEI (~1.8 LMH/bar), it achieved the highest rejection of NaCl, with a value of around 90%. Having a relatively high value of water permeability (~13.2 LMH/bar) and a relatively low value of NaCl rejection (~24%) manifest the imperfect IP layer formed on the PAN-A substrate modified with PDADMAC. In addition to the imperfection, the characterization results also imply that less part of the IP layer peeled off from the surface modified with PDADMAC, since the water permeability is markedly lower than that for the PAN-A substrate. A summary of recent studies focusing on fabricating TFC membranes based on PAN substrates via IP is presented in Table S1.

As revealed by the contact angle characterization in Figure 1, the alkaline treatment significantly increased the hydrophilicity of the polymeric network of the PAN; the deposition of PAH onto the surface of the PAN-A substrate then enabled the formation of an IP layer without delamination. However, it remains unclear if the employment of a hydrophilic substrate would offer any advantages in comparison with the relatively hydrophobic substrate (i.e., the PAN-O substrate), since the role of the substrate for a TFC membrane is usually negligible when evaluated using a pressure-driven process. Therefore, both the TFC membranes with the PAN-O substrate and the PAN-A substrate modified with PAH were further characterized using FO processes, where the driving force was the osmotic pressure difference, and the performance should be strongly dependent on the substrate [54].

**Figure 12:**
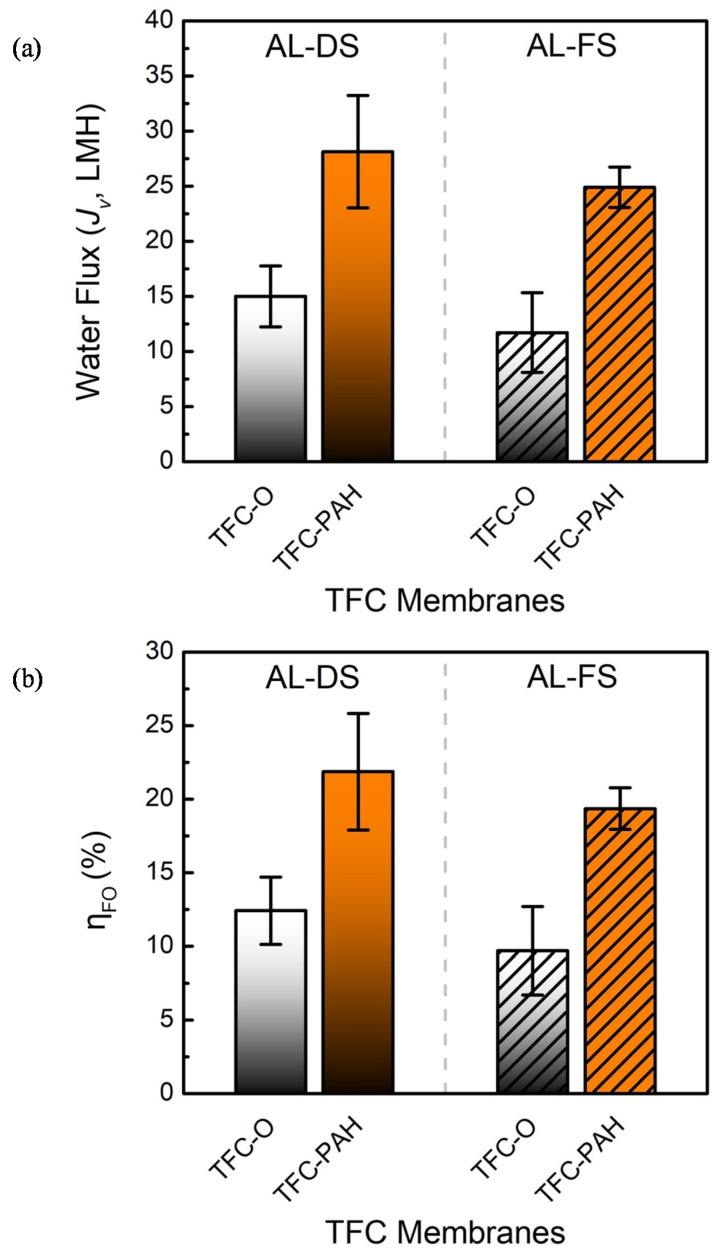
Comparison of the FO performance of the TFC membranes with the PAN-O substrate and the PAN-A substrate modified with the deposition of PAH: (**a**) the water flux and (**b**) the FO efficiency (i.e., the ratio of the measured water flux to that for the case ignoring any concentration polarization). An aqueous solution of 2 M NaCl solution was employed as the draw solution, whereas the feed solution was Milli-Q water; the active layer (i.e., the IP layer) was exposed to the feed solution (i.e., the orientation of AL-FS) or the draw solution (i.e., the orientation of AL-DS). The error bars correspond to the standard deviations of the three independent measurements.

The water flux for each of the TFC membranes was measured during the FO processes with two different membrane orientations; that is, the active layer was exposed to the draw solution (denoted by AL-DS) or the feed solution (denoted by AL-FS). It was revealed by prior studies [55] that the dilutive ICP should result in the greater loss of the driving force when implementing the FO process with the AL-FS orientation. Despite the difference resulting from the membrane orientation, the TFC-PAH yielded a water flux of approximately 25~28 LMH when employing a draw solution of 2 M NaCl, which was nearly twofold greater than the water flux of approximately 12~15 LMH for the TFC-O, as shown in Figure 12a. However, the TFC-O and TFC-PAH had similar values in terms of the water permeability (~1.2 LMH/bar and ~1.3 LMH/bar, respectively) determined in the pressure-driven processes. Moreover, the comparison of the SEM images in Figure 2 supports the hypothesis that the alkaline treatment should have a marginal impact on the geometrical characteristics of the PAN substrate. Therefore, it is reasonable to infer that the wettability of the substrate network could have a substantial impact on the ICP.

The effect of the substrate on the FO performance can be further quantified by calculating the FO efficiency (*η_FO_*), which is the ratio of the measured water flux to that for the ideal case, ignoring the CP phenomena (i.e., A·Δπ) [56]. The calculation results are shown and compared in Figure 12b. It is evident that the FO efficiency of the TFC-PAH was almost doubled in comparison with that for the TFC-O. Note that, in terms of the classical FO model [55], the FO efficiency is also a function of the characteristic concentration determined by the intrinsic properties of the active layer, which can be evaluated as the ratio of the solute flux to the water flux. The TFC-PAH gave rise to a value of *J_s_/ J_V_* (3.5 ± 0.8 g/L), which is very close to that for the TFC-O (3.7 ± 0.5 g/L), indicating that the difference in the FO efficiency could be mainly attributed to the substrate modified with the alkaline treatment.

Although all of the characterization results are in good agreement with the hypothesis that increasing the wettability of the substrate could favor the enhancement of the FO efficiency, it is quite challenging to unravel the mechanisms accounting for the complex transport phenomena in the substrate, especially when the effects of the wettability of the polymeric network cannot be ignored. A possible explanation is that the variation in the wettability could significantly change the topological substructures of the network [57] and thereby give rise to different paths that are effective for the fluid flow and the solute flux. More work will be carried out to further explore the role of the wettability of the substrate during FO processes.

## 4. Conclusions

The effects of the PAN substrate on the IP-film formation were investigated while highlighting the role of the surface hydrophilicity. The surface of the PAN substrate modified with the alkaline treatment was tuned by the deposition of different polyelectrolytes to vary the degree of the hydrophilicity. All of the modified PAN substrates were employed to fabricate the TFC membranes via IP, and the IP layers formed were characterized and compared in order to correlate the film formation with the degree of hydrophilicity. The following conclusions can be drawn from the characterization results:

i. The PAN substrate with a relatively hydrophilic surface could result in delamination when it is employed in the formation of the TFC membrane via IP, whereas the integrity of the IP layer can be improved by decreasing the surface hydrophilicity.
ii. The deposition of various polycations onto the surface of the PAN substrate modified with the alkaline treatment can change the wettability to different degrees (from ~38 to ~93 degrees), thereby offering a tool for the IP-based fabrication of TFC membranes with a hydrophilic PAN substrate.
iii. The comparative study of the evaluation of the TFC membranes with different PAN substrates indicated that a relatively hydrophilic substrate would favor the enhancement of the water–flux efficiency with a factor of ~2 when it was employed in an osmotically-driven process.

All of the modified PAN substrates were also examined using SEM and AFM; the characterization results confirmed that the variations in the geometrical characteristics were negligible. Therefore, it is reasonable to believe that the effects of the modified PAN substrates on the IP-film formation were primarily attributed to the changes in the surface wettability. Although this study shed light on the role of the substrate hydrophilicity in a membrane-supported IP process, the performance of the fabricated TFC membranes needs to be further enhanced before being adopted in industrial sectors. The application of polyelectrolyte deposition not only provided an approach to the investigation of the effects of the surface hydrophilicity but also opened a new paradigm for the optimization of the fabrication of TFC membranes via IP.

## Figures and Tables

**Table 1:** Modification conditions for the PAN substrates and the TFC membranes fabricated via IP.

	Abbreviations	Modification Conditions
PAN substrates	PAN-O	original PAN substrate
	PAN-H	PAN-O + heat treatment ^a^
	PAN-A	PAN-O + alkaline treatment ^b^
	PDADMAC-m	PAN-A + deposition of PDADMAC ^c^
	PEI-m	PAN-A + deposition of PEI
	PAH-m	PAN-A + deposition of PAH
TFC membranes	TFC-O	PAN-O + IP ^d^
	TFC-H	PAN-H + IP
	TFC-A	PAN-A + IP
	TFC-PDADMAC	PDADMAC-m + IP
	TFC-PEI	PEI-m + IP
	TFC-PAH	PAH-m + IP

^a^ The PAN substrate was immersed in Milli-Q water at a temperature of 45 °C for 90 min. ^b^ The PAN substrate was immersed in an aqueous solution of 1.5 M NaOH at a temperature of 45 °C for 90 min. ^c^ The dense surface was exposed to an aqueous solution containing 1 g of the polyelectrolyte and 0.5 M NaCl for 20 min. ^d^ The IP was implemented by contacting the PAN substrate (impregnated with an the aqueous solution of 2.0 wt.% MPD) with the organic solution (0.1 *w*/*v*.% TMC dissolved in hexane).

## Supplementary Materials

The following are available online at https://www.mdpi.com/2077-0375/10/10/259/s1, Table S1: Recent studies (form 2018 to 2020) of TFC membranes fabricated based on PAN substrates via IP; Figure S1: Enlarged SEM images of the original PAN substrate and the PAN substrates with the heat treatment and alkaline treatment; (a) The enlarged SEM image of the original PAN substrate (i.e., PAN-O); (b) The enlarged SEM image of the PAN substrate with the heat treatment (i.e., PAN-H); (c) The enlarged SEM image of the PAN substrate with the alkaline treatment (i.e., PAN-A).
