# Supplementary material for: Effects of the Substrate on Interfacial Polymerization: Tuning the Hydrophobicity via Polyelectrolyte Deposition
Source: Membranes (Basel). 2020 Sep 26;10(10):259. doi: 10.3390/membranes10100259 (PMC7599731; doi:10.3390/membranes10100259)
Supplement: Supplementary file 1 [file membranes-10-00259-s001.pdf]

membranes

Supplementary Materials: Effects of the Substrate on Interfacial Polymerization: Tuning the Hydrophobicity via Polyelectrolyte Deposition

Xin Liu [1,†], Ge Liu [1,†], Weiyi Li [1,*], Qinyu Wang [1] and Baolin Deng [2]

1 School of Environmental Science and Engineering, Southern University of Science and Technology, Shenzhen 518055, China; liux5@sustech.edu.cn (X.L.); 11749132@mail.sustech.edu.cn (G.L.); qwang437@gatech.edu (Q.W.)
2 Department of Civil and Environmental Engineering, University of Missouri, Columbia, MO 65211, USA; DengB@missouri.edu (B.D.)
* Correspondence: liwy3@sustech.edu.cn (W.L.); Tel.: +86-755-8801-0821
† These authors have made equal contribution to the current study.

Received: 24 August 2020; Accepted: 21 September 2020; Published: 26 September 2020

Table S1. Recent studies (form 2018 to 2020) of TFC membranes fabricated based on PAN substrates via IP.

TFC Membranes	Fabrication Conditions	Water Permeability	NaCl Rejection	Reference
TFC-PAN	nanofibrous PAN substrate MPD and TMC for IP	N.A.	0 (intact PA layer)	Peng L. E. *et al.*[1]
NF-1	PAN substrate PIP and TMC for IP	41.8 LMH @ 10 bar	44%	Polisetti V. *et al.*[2]
NF-2	PAN substrate PIP and TMC for IP with 0.01% PSS	69 LMH @ 10 bar	40%	
NF-3	PAN substrate PIP and TMC for IP with 0.1% PSS	90 LMH @ 10 bar	35%	
NF-4	PAN substrate PIP and TMC for IP with 0.5% PSS	203 LMH @ 10 bar	~30%	
PA20/PAN TFNC	PAN substrate PIP and TMC for IP with 1% TEA and 20% NaCl	~185 LMH @ 5 bar	26%	Shen K. *et al.*[3]
TFC-10%	hydrolyzed double-skinned PAN (10%) substrate MPD and TMC for IP	1.99 LMH/bar	87.9%	He M. *et al.*[4]
TFC-s	single-skinned PAN substrate MPD and TMC for IP	1.44 LMH/bar	84.7%	
PIP-Z/PAN	PAN substrate PIP and TMC for IP PEI-SBMA coating	8.6 LMH/bar	64.5%	Chiao Y.-H. *et al.*[5]
ICIC/ZnO/HBPA membrane	PAN substrate ICIC and HBPA for IP with ZnO NPs	53 LMH @ 6 bar	60.8%	Zhang X. *et al.*[6]
PA/PAN	PAN substrate PIP and TMC for IP	~120 LMH @10 bar	~17%	Wu M. *et al.*[7]

PA/PDA/PAN	PAN substrate<br>PIP and TMC for IP<br>with PDA interlayer	~163 LMH @10 bar	~15%	
PA/PDA-COF(3)/PAN	PAN substrate<br>PIP and TMC for IP<br>with PDA-COF interlayer	~207 LMH @10 bar	~20%	
TFC	PAN substrate<br>PIP and TMC for IP	6.89 LMH/bar	~28-44%	Liu H. *et al.*[8]
TFN-0.05	PAN substrate<br>PIP and TMC for IP<br>with 0.05% UiO-66-NH2	12.68 LMH/bar	~35-40%	
TFN-0.1	PAN substrate<br>PIP and TMC for IP<br>with 0.10% UiO-66-NH2	14.55 LMH/bar	~34-38%	
TFN-0.15	PAN substrate<br>PIP and TMC for IP<br>with 0.15% UiO-66-NH2	13.13 LMH/bar	~30-35%	
HNT/PA NF	PAN substrate<br>DETA and TMC for IP<br>with HNT	14.5 LMH @ 6 bar	62.8%	Yan L. *et al.*[9]
TFC-TIP	PAN substrate<br>MPD and TMC for IP<br>TMC in different organic solvents	3.26 LMH/bar	98.1%	Kwon H. *et al.*[10]
TFC-HIP	PAN substrate<br>MPD and TMC for IP<br>TMC in toluene	0.79 LMH/bar	94.5%	
HTI-TFC	commercial HTI-TFC	1.48 LMH/bar	94.5%	
TFC-CS-PAN-1	PAN nanofibers<br>CS-GA modification<br>MPD and TMC for IP	6.4 LMH/bar	46.5%	Chi X.-Y. *et al.* [11]
TFC-CS-PAN-2	PAN nanofibers<br>CS-GA modification<br>MPD and TMC for IP	4.2 LMH/bar	59.9%	
TFC-CS-PAN-3	PAN nanofibers<br>CS-GA modification<br>MPD and TMC for IP	2.8 LMH/bar	66.0%	
TFC-CS-PAN-4	PAN nanofibers<br>CS-GA modification<br>MPD and TMC for IP	1.6 LMH/bar	83.5%	
TFC	PAN substrate<br>PIP and TMC for IP	106 LMH @ 13 bar	25 %	Esmaeili M. *et al.*[12]
SW30HR	commercial SW30HR	1.78×10^{-12} $m \cdot Pa^{-1} \cdot s^{-1}$	97.5%	Li M. *et al.*[13]
TFN-H	commercial PAN membrane as substrate<br>MPD and TMC for IP<br>surface modified imogolite nanotubes with n-hexane	1.47×10^{-12} $m \cdot Pa^{-1} \cdot s^{-1}$	95.2%	
TFN-H1		1.92×10^{-12} $m \cdot Pa^{-1} \cdot s^{-1}$	96.1%	
TFN-H2		2.24×10^{-12} $m \cdot Pa^{-1} \cdot s^{-1}$	96.4%	
TFN-H3		2.61×10^{-12}	96.1%	

		$m \cdot Pa^{-1} \cdot s^{-1}$	
TFN-H4		2.24×10^{-12} $m \cdot Pa^{-1} \cdot s^{-1}$	96.8%
TFN-H5		2.91×10^{-12} $m \cdot Pa^{-1} \cdot s^{-1}$	96.8%
TFN-H6		3.53×10^{-12} $m \cdot Pa^{-1} \cdot s^{-1}$	96.3%
TFN-C	commercial PAN membrane as substrate	1.10×10^{-12} $m \cdot Pa^{-1} \cdot s^{-1}$	13.6%
TFN-C1	MPD and TMC for IP	2.01×10^{-12} $m \cdot Pa^{-1} \cdot s^{-1}$	13.3%
TFN-C2	surface modified imogolite nanotubes with chloroform	3.39×10^{-12} $m \cdot Pa^{-1} \cdot s^{-1}$	17.2%

(a)

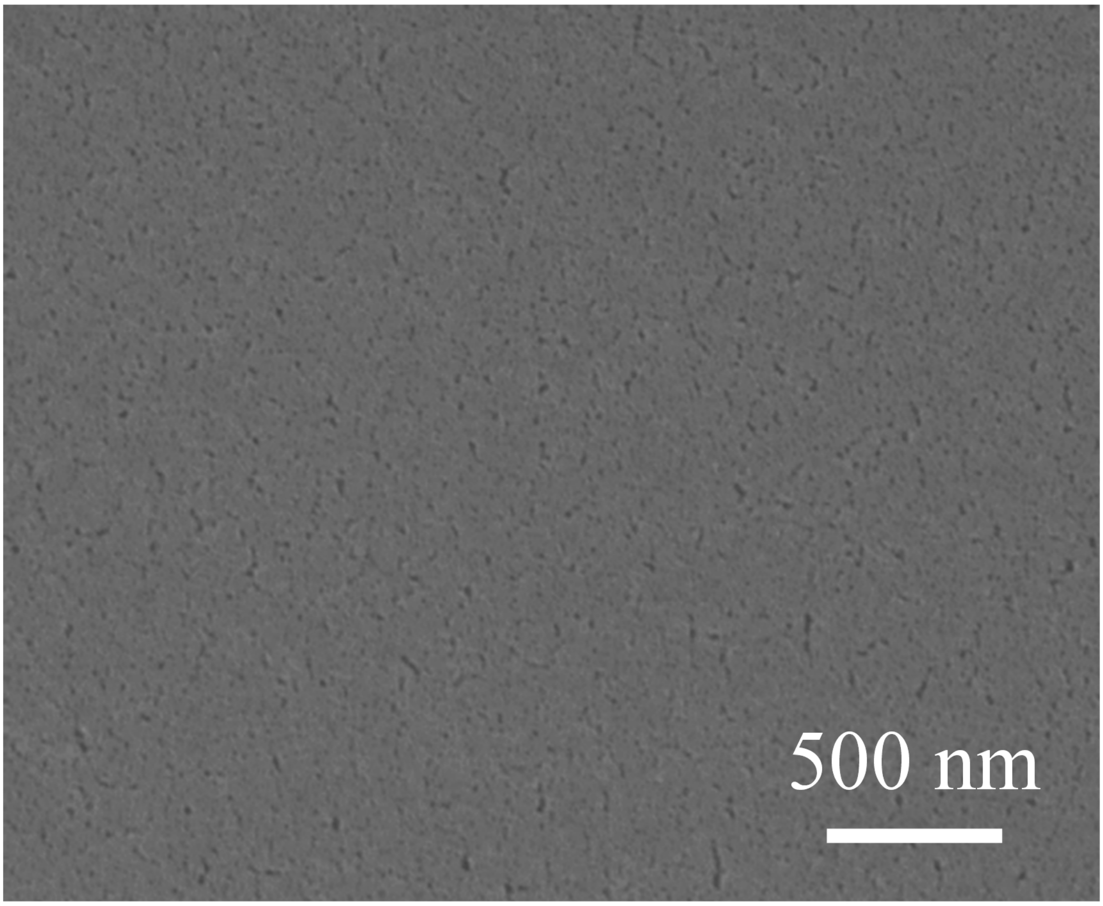

(b)

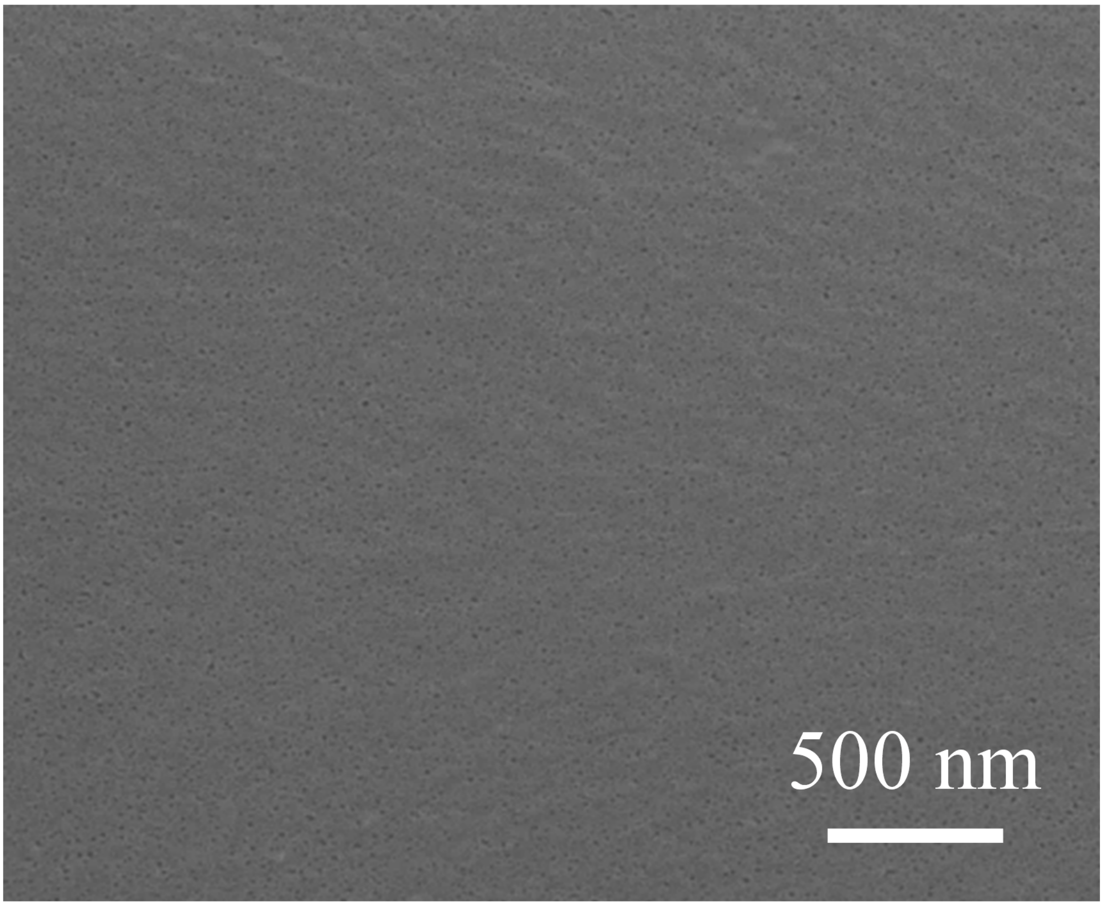

(c)

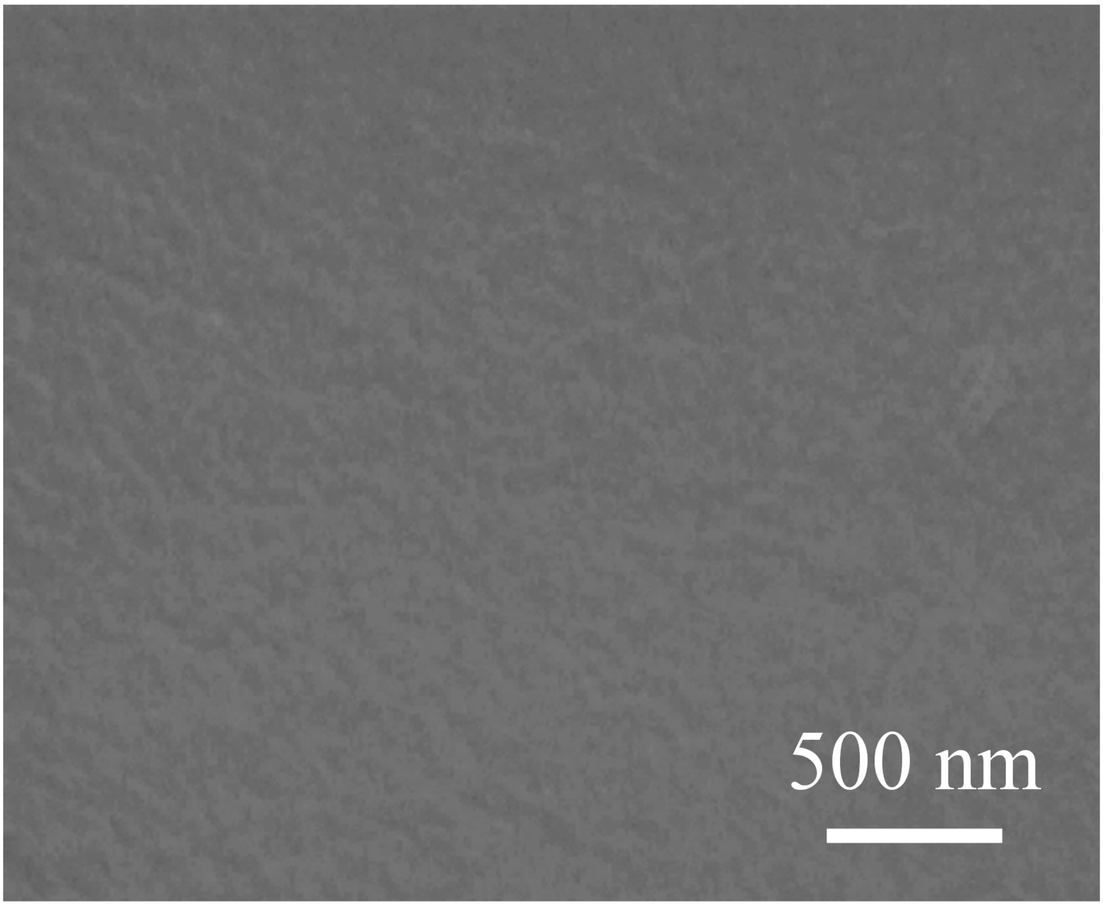

Figure 1. Enlarged SEM images of the original PAN substrate and the PAN substrates with the heat treatment and alkaline treatment; (**a**) The enlarged SEM image of the original PAN substrate (i.e., PAN-O); (**b**) The enlarged SEM image of the PAN substrate with the heat treatment (i.e., PAN-H); (**c**) The enlarged SEM image of the PAN substrate with the alkaline treatment (i.e., PAN-A).

References

1. Peng, L.E.; Yao, Z.; Yang, Z.; Guo, H.; Tang, C.Y. Dissecting the Role of Substrate on the Morphology and Separation Properties of Thin Film Composite Polyamide Membranes: Seeing Is Believing. *Environmental Science & Technology* **2020**, *54*, 6978-6986.
2. Polisetti, V.; Ray, P. Thin film composite nanofiltration membranes with polystyrene sodium sulfonate-polypiperazinetrimesamide semi-interpenetrating polymer network active layer. *Journal of Applied Polymer Science* **2020**, *137*.
3. Shen, K.; Li, P.; Zhang, T.; Wang, X. Salt-tuned fabrication of novel polyamide composite nanofiltration membranes with three-dimensional turing structures for effective desalination. *Journal of Membrane Science* **2020**, *607*
4. He, M.; Wang, L.; Lv, Y.; Wang, X.; Zhang, Z.; Cui, Q.; Zhu, J. Effect of a novel hydrophilic double-skinned support layer on improving anti-fouling performance of thin-film composite forward osmosis membrane. *Colloids and Surfaces a-Physicochemical and Engineering Aspects* **2020**, *602*.
5. Chiao, Y.-H.; Patra, T.; Ang, M.B.M.Y.; Chen, S.-T.; Almodovar, J.; Qian, X.; Wickramasinghe, S.R.; Hung, W.-S.; Huang, S.-H.; Chang, Y., et al. Zwitterion Co-Polymer PEI-SBMA Nanofiltration Membrane Modified by Fast Second Interfacial Polymerization. *Polymers* **2020**, *12*.
6. Zhang, X.; Tang, S. The New Method for Measuring Internal Stress of Polymer Products. *China Plastics Industry* **2020**, *48*, 91-94.
7. Wu, M.; Yuan, J.; Wu, H.; Su, Y.; Yang, H.; You, X.; Zhang, R.; He, X.; Khan, N.A.; Kasher, R., et al. Ultrathin nanofiltration membrane with polydopamine-covalent organic framework interlayer for enhanced permeability and structural stability. *Journal of Membrane Science* **2019**, *576*, 131-141.

8. Liu, H.; Gao, J.; Liu, G.; Zhang, M.; Jiang, Y. Enhancing Permeability of Thin Film Nanocomposite Membranes via Covalent Linking of Polyamide with the Incorporated Metal-Organic Frameworks. *Industrial & Engineering Chemistry Research* **2019**, *58*, 8772-8783.
9. Yan, L.; Liu, Y.; Deng, H.; Chen, Q.; Duan, L.; Liu, X.; Bang, Y.; Shi, J.; Na, B. Fabrication of halloysite nanotube/polyamide nanofiltration membranes via interfacial polymerization. *Modern Chemical Industry* **2019**, *39*, 68-72, 76.
10. Kwon, H.-E.; Kwon, S.J.; Park, S.-J.; Shin, M.G.; Park, S.-H.; Park, M.S.; Park, H.; Lee, J.-H. High performance polyacrylonitrile-supported forward osmosis membranes prepared via aromatic solvent-based interfacial polymerization. *Separation and Purification Technology* **2019**, *212*, 449-457.
11. Chi, X.-Y.; Xia, B.-G.; Xu, Z.-L.; Zhang, M.-X. Impact of Cross-Linked Chitosan Sublayer Structure on the Performance of TFC FO PAN Nanofiber Membranes. *Acs Omega* **2018**, *3*, 13009-13019.
12. Esmaeili, M.; Mansoorian, S.H.; Gheshlaghi, A.; Rekabdar, F. Performance and Morphology Evaluation of Thin Film Composite Polyacrylonitrile/Polyamide Nanofiltration Membranes Considering the Reaction Time. *Journal of Water Chemistry and Technology* **2018**, *40*, 219-227.
13. Li, M.; Brant, J.A. Synthesis of polyamide thin-film nanocomposite membranes using surface modified imogolite nanotubes. *Journal of Membrane Science* **2018**, *563*, 664-675.

© 2020 by the authors. Licensee MDPI, Basel, Switzerland. This article is an open access article distributed under the terms and conditions of the Creative Commons Attribution (CC BY) license (http://creativecommons.org/licenses/by/4.0/).
